# Bonding to Different PEEK Compositions: The Impact of Dental Light Curing Units

**DOI:** 10.3390/ma10010067

**Published:** 2017-01-14

**Authors:** Nina Lümkemann, Marlis Eichberger, Bogna Stawarczyk

**Affiliations:** Department of Prosthodontics, Dental School, Ludwig-Maximilians-University Munich, Goethestrasse 70, 80336 Munich, Germany; nina.luemkemann@med.uni-muenchen.de (N.L.); marlis.eichberger@med.uni-muenchen.de (M.E.)

**Keywords:** PEEK, TiO_2_, adhesive system, light curing units, LED, halogen, bonding properties, tensile bond strength, fracture types

## Abstract

This study investigated the impact of different light curing units (LCUs) for the polymerization of adhesive system visio.link (VL) on the tensile bond strength (TBS) of different PEEK compositions. For TBS measurements, 216 PEEK specimens with varying amounts of TiO_2_ (PEEK/0%, PEEK/20%, PEEK/>30%) were embedded, polished, air abraded (Al_2_O_3_, 50 µm, 0.4 MPa), conditioned using VL, and polymerized using either a halogen LCU (HAL-LCU) or a LED LCU (LED-LCU) for chairside or labside application, respectively. After thermocycling (5000×, 5/55 °C), TBS was measured, and fracture types were determined. Data was analyzed using a 2-way ANOVA followed by Tukey–HSD, Kruskal–Wallis H and Mann–Whitney U tests as well as a Chi^2^-test and a Ciba–Geigy table (*p* < 0.05). Globally, the light curing units, followed by PEEK composition, was shown to have the highest impact on TBS. The HAL-LCUs, compared to the LED-LCUs, resulted in a higher TBS for all PEEK compositions—without significant differences between chairside and labside units. Regarding the different PEEK compositions, PEEK/20%, compared to PEEK/0%, resulted in a higher TBS when both, HAL-LCUs or LED-LCUs were used for labside application. In comparison with PEEK/>30%, PEEK/20% resulted in a higher TBS after using HAL-LCU for labside application. No significant differences were found between PEEK/0% and PEEK/>30%. HAL-LCU with PEEK/20% for labside application showed a higher TBS than HAL-LCU with PEEK/20% for chairside application, whereas LED-LCU with PEEK/>30% for chairside application showed a higher TBS than LED-LCU with PEEK/>30% for labside application.

## 1. Introduction

In dentistry, polyetheretherketone (PEEK) is one of the most frequently used high-performance thermoplastic [1]. Due to its excellent mechanical, physical, and chemical properties [2,3] it is applicable in a wide range of indications in the dental field [4]. Even though PEEK is a plastic material with a low elastic modulus, the mechanical properties are adjustable by adding varying amounts of titanium oxide (TiO_2_) as filler particles [5]. TiO_2_ is already known for different applications in dentistry, and, similar to PEEK, it is proven to be biocompatible. In addition, adding TiO_2_ as inorganic filler particles also satisfies the optical properties for dental applications with PEEK and endorses extending the range of indications. For this reason, many studies have investigated the material regarding the relevant properties, e.g., wear resistance [6], fracture load [7,8,9], retention load and retention forces [10,11], hardness [12], flexural behavior [13], and discoloration [14]. PEEK has thus been proven to be a suitable material for fixed dental prostheses (FDPs) [15,16,17] and long-term restorations [4,6,10,11,12,13,18].

However, to apply PEEK successfully to the applications mentioned above, a durable and effective bonding to other dental materials is required. However, since PEEK is in focus in the field of dental materials science, this demand is known to be a critical factor due to its inert surface character. Recent studies have proven the enhanced bonding properties via surface modifications with PEEK consisting of air-particle abrasion and/or conditioning using adhesive systems. Regarding the particle size of Al_2_O_3_, no influence on bonding properties has been found [18]. Regarding adhesive systems, compositions based on methylmethacrylate (MMA) in combination with other dimethacrylates (DMAs) showed significantly higher bonding strength [19].

Internal investigations indicated that the adhesive system Pekk Bond (Anaxdent) resulted in a significant higher bond strength when the PEEK surface was air-abraded with higher pressure (0.4 MPa). In comparison, the adhesive system visio.link (bredent) resulted in the highest bond strength irrespectively of the air-abrasion pressure when it was applied as recommended by the manufacturer. Since visio.link has been the most frequently investigated adhesive system on PEEK resulting in the highest bond strength [12,20,21,22,23,24], the chemical mechanism of adhesion is still challenging. Successful bonding is known to be a complex phenomenon that depends on many parameters and the interaction of chemical, physical, and mechanical effects can be influenced in unexpected ways by changing only one parameter during the bonding process.

With regard to the high potential of different PEEK compositions—unfilled and filled with varying amounts of TiO_2_ particles—it is desirable to enable a durable cementation of PEEK restorations in chairside applications. Due to new technologies, chairside applications have become more and more attractive for the patient and the dentist, as it is timesaving. For this, LED light curing units (LED-LCUs) have been developed to replace the common halogen light curing units (HAL-LCUs). In comparison with well-established HAL-LCUs, the newer LED-LCUs are characterized by a smaller wavelength range, higher light intensities, and a longer lifetime. Moreover, they are timesaving, more user-friendly, and more comfortable for the patient because no ventilation is required, which in turn reduces heat and noise development. However, it has to be considered that the choice of LCU has to be made taking into account the photoinitiator used in the relevant adhesive system.

Since the light curing units for the polymerization of adhesive systems as well as the varying compositions of PEEK material are possible parameters that may influence bonding properties, this study investigated the null hypotheses that neither different PEEK compositions (PEEK/0%, PEEK/20%, and PEEK/>30%) nor the application of different light curing units (LED-LCUs and HAL-LCUs designed for either chairside or labside application) affect bonding properties with respect to the adhesive system visio.link. To characterize the bonding properties, tensile bond strength was measured, and corresponding fracture types were determined.

## 2. Material and Methods

**Figure 1:**
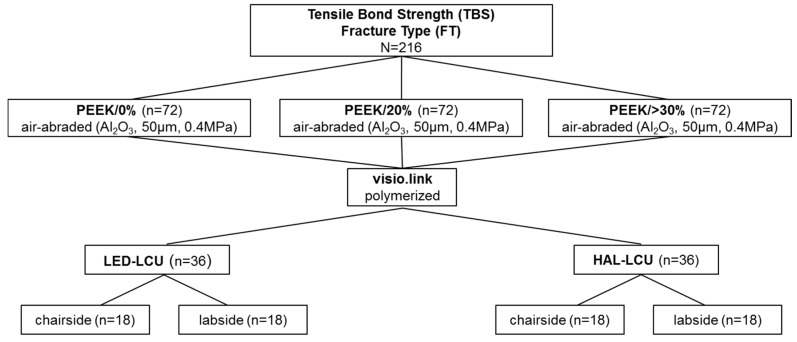
Study design for TBS measurement and fracture type analysis.

This study investigated the impact of four different light curing units (LCUs) on the effectiveness of the adhesive system visio.link (bredent, Senden, Germany) as a surface conditioner for three different PEEK compositions with varying filler amounts of TiO_2_ (PEEK/0%, PEEK/20%, PEEK/<30%) to achieve durable bonding. Therefore, tensile bond strength (TBS) measurements with fracture type (FT) analysis were performed (Figure 1). All materials and light curing units used in this study are listed in Table 1.

### 2.1. Preparation of Specimens

For TBS measurements, 216 PEEK specimens (PEEK/0%, PEEK/20%, PEEK/>30%, *n* = 72) with a surface area of approximately 16 mm^2^ were cut under dry conditions using a handpiece (KaVo EWL K9, KaVo Dental GmbH, Biberach, Germany) and embedded in acrylic resin (ScandiQuick A and B, ScanDia, Hagen, Germany, Lot No. 09201 and 09202). Specimens were polished up to P1200 (SiC Foil, Struers, Ballerup, Denmark) for 20 s with an automatic polishing device (Tegramin 20, Struers, Ballerup, Denmark) under permanent water cooling. After ultrasonically cleaning (L&R Transistor/Ultrasonic T-14, L&R, Kearny, NJ, USA) for 60 s in distilled water, the specimens were air-dried and air-abraded (basis Quattro IS, Renfert, Hilzingen, Germany) with alumina particles (Al_2_O_3_, Orbis Dental, Münster, Germany) using the following air-abrading parameters: a particle size of 50 µm, a duration of 10 s, a distance of 5 mm at 45°, and a pressure of 0.4 MPa. After air-abrasion, specimens were ultrasonically cleaned and carefully air-dried.

Seventy-two specimens for each PEEK composition were subsequently divided into 4 groups according to the type of LCU (i.e., halogen vs. LED) and the type of application (i.e., chairside vs. labside) (*n* = 18) (Figure 1, Table 1). The air-abraded surface of the PEEK specimens was conditioned for 5 s with the adhesive system visio.link (VL) using a microbrush. Then, VL was polymerized either for 90 s when HAL-LCUs were used or 10 s when LED-LCUs were used.

The HAL-LCUs and LED-LCUs differ regarding chairside and labside applications. Chairside devices are designed to be applied intraoral by the dentist. For this, the chairside LCUs have a handle and can directly and be precisely directed towards the relevant area. The light intensities for both chairside LCUs were determined (Table 1) using a precise dental radiometer (Bluephase Meter II, Ivoclar Vivadent, Schaan, Liechtenstein). In contrast, labside LCUs devices are designed for the fabrication of dental restorations in the dental laboratory. For this, so-called polymerization furnaces are used where the restoration is placed into it and interior mirrors guarantee a reflection of light onto the restoration.

The polymerization times were chosen depending on the type of LCU. The polymerization time of 90 s for the adhesive system visio.link using the halogen LCUs was chosen based on the manufacturer’s recommendation. In comparison, the reduced polymerization time of 10 s using the LED LCUs was chosen based on the commonly used adhesive systems for chairside treatments that are polymerized for 10–20 s.

**Figure 2:**
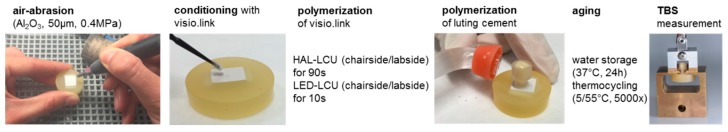
Process of specimen preparation.

After polymerization of visio.link, an acrylic cylinder (SD Mechatronik GmbH, Feldkirchen–Westerham, Germany) with an inner diameter of 2.9 mm was positioned onto the conditioned PEEK surface, filled with self-adhesive resin cement (Clearfil SA Cement, Kuraray Medical, Tokyo, Japan) and polymerized for 20 s using a chairside LED-LCU (Elipar S10, 3M, Seefeld, Germany). Subsequently, all specimens were stored in distilled water at 37 °C for 24 h (HeraCee 150, Heraeus, Hanau, Germany) and aged by thermal cycling for 5000 cycles between 5 and 55 °C (Thermocycler THE 1100, SD Mechatronik, Feldkirchen-Westerham, Germany). After 2 h of relaxation, TBS measurements were performed with a crosshead speed of 5 mm/min by applying the tensile stress perpendicular to the specimen’s surface until fracture of each specimen occurred (Zwick 1445 RetroLine, Zwick Roel Group, Ulm, Germany). TBS was calculated as the maximal force at the debonding/bonding area. Figure 2 presents the processing of the specimen preparation.

### 2.2. Fracture Type Anaysis

Corresponding FT of debonded areas were analyzed using a stereomicroscope at a magnification of 20× (Carl Zeiss Axioskop 2 MAT, Zeiss, Jena, Germany) and defined as follows: (1) adhesively—with no resin cements remnants left on the PEEK surface; (2) cohesively—with partial remnants of resin cement on PEEK to which the PEEK surface is also exposed.

### 2.3. Statistical Analyses

In the first step, data were analyzed using descriptive statistics (mean, standard deviation (SD), 95% confidence intervals (CI), minimum, median, and maximum). A Kolmogorov–Smirnov test examined the test groups on the assumption of normality. For global analysis, a univariate 2-way ANOVA was calculated followed by a partial eta-squared (η_P_^2^) to determine the effects of the investigated parameters on the results of TBS. Moreover, significant differences and homogenous groups were determined using a post-hoc Tukey–HSD test. Data were divided according to the null hypothesis and additional analyzed using non-parametric Kruskal–Wallis H and Mann–Whitney U tests. The relative frequency of fracture types were analyzed according to a Chi^2^-test and a Ciba–Geigy table. The level of significance was set to 5% for all statistical tests. All analyses were computed using the software IBM SPSS (Version 23; IBM Corporation, Armonk, NY, USA).

## 3. Results

### 3.1. Tensile Bond Strength

The highest impact on TBS was exerted by the light curing units (η_P_^2^ = 0.630, *p* < 0.001), while the PEEK composition also affected the TBS (η_P_^2^ = 0.055, *p* = 0.003). The Kolmogorov–Smirnov test indicated that 25% of the test groups were not normally distributed (Table 2). Therefore, the data was analyzed non-parametrically. The global analysis indicated significant higher TBS values for chairside and labside HAL-LCUs compared to the chairside and labside LED-LCUs (*p* = 0.003). However, between the HAL-LCUs and LED-LCUs, no significant differences regarding the intended individual application that the light units are designed for (chairside or labside) were observed. Regarding the different PEEK compositions, PEEK/20% showed the highest results of TBS (*p* < 0.001) followed by PEEK/>30% and PEEK/0% (Table 2).

**Figure 3:**
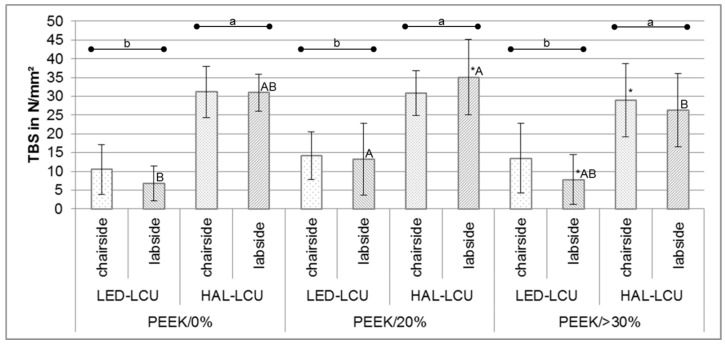
TBS (mean ± SD) divided by different PEEK compositions and LCUs with significant differences, respectively.

After dividing data according to null hypotheses, non-parametric tests indicated significant differences between PEEK compositions with HAL-LCU and LED-LCU for labside application (*p* < 0.022) (Table 2). Compared to PEEK/0%, PEEK/20% resulted in a higher TBS when labside LED-LCU were applied (*p* < 0.034). Compared to PEEK/>30%, PEEK/20% resulted in a higher TBS when labside HAL-LCU was applied (*p* < 0.01). In comparison with PEEK/0% and PEEK/>30%, no significant differences were found with respect to the LCUs (*p* > 0.181). Regarding the LCUs, significant differences between PEEK/20% and PEEK/>30% were found (*p* < 0.001). For PEEK/20%, the labside HAL-LCU resulted in a higher TBS than the chairside halogen unit (*p* = 0.007), while for PEEK/>30%, the chairside LED-LCU reached higher values than the labside LED-LCU (*p* = 0.047) (Table 2, Figure 3).

### 3.2. Fracture Types

The fracture types showed significant differences between the different LCUs (*p* < 0.001). The adhesive fracture type occurred more frequently for specimens polymerized using LED-LCUs, while for specimens polymerized with HAL-LCUs, the cohesive fracture type was observed more frequently (Table 3). No differences in fracture type were found between PEEK compositions. (*p* = 0.878).

## 4. Discussion

Previously published literature has proven the adhesive system visio.link to achieve the best bonding properties as a surface conditioner after pretreatment of PEEK specimens when applied as recommended by the manufacturer. To the best of our knowledge, the impact of different light curing units for polymerization of visio.link on the bonding strength in combination with different PEEK compositions has not been investigated yet.

**Figure 4:**
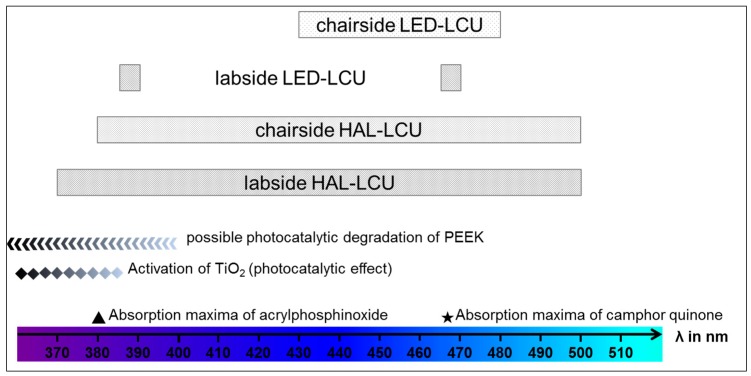
Summary of the possible parameters and effects that may influence the bonding properties to different PEEK compositions with respect to the adhesive system visio.link that was polymerized with four different LCUs and their corresponding wavelengths.

First of all, the results show that the highest bond strength for all PEEK compositions was achieved after polymerizing visio.link using halogen LCUs for both chairside or labside application. On the one hand, this is in accordance with all previously published studies [12,20,21,22,23,24]. On the other hand—of course—this is in accordance with the recommendation of the manufacturer. Basically, this fact is based on the chemical composition of visio.link as the used photoinitiator (diphenyl(2,4,6,-trimethylbenzoyl)phosphinoxide) requires a certain wavelength to cure successfully. The photoinitiator acrylphosphinoxide is a commonly used system for dental materials and shows the corresponding absorption maximum at a wavelength of 380 nm. The results and technical details show that the certain range of wavelength is only provided by the halogen LCUs but not by the LED-LCUs (Figure 4). Alternatively, to acrylphosphinoxide, camphor quinone is another well-established photoinitiator often used in dentistry. Compared to acrylphosphinoxide, camphor quinone shows the absorption maximum at higher wavelengths (468 nm) (Figure 4). This range of wavelength in turn is provided by the LED-LCUs but also by the HAL-LCUs and shows that HAL-LCUs provide a wider range of wavelength and thus are applicable for curing different photoinitiator systems successfully.

Moreover, the results show that the bonding properties are affected by the PEEK composition, as the amount of 20% TiO_2_ filler particles resulted in the highest bond strength. Regarding the halogen LCUs, this finding may be caused by the activation of TiO_2_ particles at wavelengths smaller than 385 nm. TiO_2_ particles are known for intense UV absorption and superior hydrophilicity [25]. For medical applications, the antibacterial characteristics of irradiated TiO_2_ materials especially are of interest [26]. When the antibacterial activity of nano-TiO_2_-reinforced PEEK/PEI blends against two bacteria was investigated, reduced survival rates of the bacteria after UV irradiation with a wavelength of 365 nm were found [5]. This effect can be associated with the generation of reactive radicals (oxygen species) when TiO_2_ materials are irradiated with UV light [27]. As the results of the present study were not explicit regarding the different PEEK compositions, it is a question whether dental LCUs provide the spectral range and fluency rates that are needed in order to achieve a significant photocatalytic effect of TiO_2_ particles at all. The result that the TBS of PEEK/>30% irradiated with the labside HAL-LCU is not comparable to PEEK/20% could be explained by the differences in the type, morphology, particle size, or possible coating of the TiO_2_ particles. Unfortunately, no information about the filler particles were provided by the manufacturer. However, with respect to the antibacterial action of TiO_2_ materials, a higher bioactivity was reported when TiO_2_ nanoparticles were used compared to conventional microparticles [28].

With respect to the present study, the generation of reactive radicals on the irradiated surface of the 20%-TiO_2_-filled PEEK composition with the halogen LCUs may improve the bonding properties to the initially inert surface character. This assumption can also be justified by a heating effect that may be greater for halogen LCUs than for LED-LCUs. This in turn can be accompanied by a higher degree of conversion of the adhesive monomers resulting in higher bond strengths. As the distance between the light source and the specimen surface varies regarding chairside or labside LCUs, this assumption should be investigated in detail using a standardized test set-up. The heating effect should be determined with respect to the PEEK composition and the LCU in combination. Moreover, a comparison of the output spectra of all investigated LCUs would be helpful in subsequent studies. In general, the measurement of the light intensities of the chairside LED-LCUs showed higher values than the chairside HAL-LCUs, which are in accordance with the manufacturer’s information.

At last, the photocatalytic degradation of PEEK is another important aspect that has to be taken into consideration when the surface is irradiated by LCUs to polymerize the transparent adhesive system visio.link. Even though PEEK is known to be resistant to radiation [2], investigations have found an impact of UV radiation (250–400 nm) on the mechanical properties of PEEK sheets resulting in reduced hardness [29]. This is comprehensible, as the photocatalytic degradation of PEEK causes chain scission reactions, crosslinking, and the formation of carbonyl and hydroxyl groups [5,30]. Certainly, in the ATR-FTIR spectrum of irradiated nano-TiO_2_-reinforced PEEK/PEI blends (356 nm), hardly any changes were found. This proves the high UV resistance of the material; nonetheless, UV radiation has also recently been used to functionalize PEEK materials [31], which in turn emphasizes the strong effect of UV radiation on PEEK surfaces once again.

## 5. Conclusions

The adhesive system visio.link achieves effective and durable bonding with different PEEK compositions when it is polymerized using a halogen LCU for 90 s. Due to the results found in this study, varying parameters such as different ranges of wavelength and the presence of TiO_2_ filler particles are assumed to cause complex effects that influence the bonding properties for PEEK conditioned with visio.link. Further research is necessary to prove and to understand the possible impacts of these effects.

## Figures and Tables

**Table 1:** Summary of used products and light curing units (LCUs).

Material Groups			Product Name Abbreviation	Manufacturer	LOT No.
**PEEK**			Tizian PEEK PEEK/0%	Schütz Dental Group, Rosbach, Germany	2014004126
			Dentokeep PEEK PEEK/20%	nt-trading, Karlsruhe, Germany	11DK14001
			bre.CAM. BioHPP dentine shade 2 PEEK/>30%	bredent, Senden, Germany	438251
**Adhesive system**			visio.link (VL)	bredent, Senden, Germany	135071
**Luting cement**			Panavia SA Cement	Kuraray Medical Inc., Tokyo, Japan	058AAA
**Light curing unit (LCU)**					**Wavelength/Light Intensity**
	**LED**	**chairside**	Elipar S10	3M, Seefeld, Germany	430–480 nm 1200 mW/cm^2^
		**labside**	EyeVolution MAX	Dreve, Unna, Germany	1 × 385−390 nm 6 × 465−470 nm
	**Halogen**	**chairside**	Translux CL	Heraeus Kulzer, Hanau, Germany	380–500 nm 450 mW/cm^2^
		**labside**	bre.Lux Power Unit	bredent, Senden, Germany	370–500 nm

**Table 2:** Descriptive statistics such as mean with standard deviation (SD), 95% confidence intervals (95% CI), and the minimum/median/maximum. All values for TBS are presented in MPa (N/mm^2^).

TBS					
PEEK	Light Curing Unit		Mean ± SD	95% CI	Min/Median/Max
PEEK/0%	LED-LCU	chair	10.5 ± 6.7 ^b^	7.0; 13.8	0.0/9.0/ 23.8
		lab	6.8 ± 4.7 ^B,b^	4.3; 9.2	0.0/6.4/14.7
	HAL-LCU	chair	31.2 ± 6.8 ^a^	27.7; 34.6	12.1/31.0/41.2
		lab	31.0 ± 4.9 ^A,B,a^	28.4; 33.5	22.3/31.6/38.6
PEEK/20%	LED-LCU	chair	14.2 ± 6.4 ^b^	10.8; 17.4	0.0/15.6/23.6
		lab	13.2 ± 9.6 ^A,b^	8.3; 18.0	0.0/10.9/35.3
	HAL-LCU	chair	30.9 ± 6.0 ^a^	27.7; 33.9	15.3/32.5/39.1
		lab	35.1 ± 10.0 *^,A,a^	30.0; 40.2	0.0/37.3/43.9
PEEK/>30%	LED-LCU	chair	13.5 ± 9.2 ^b^	8.8; 17.9	0.0/12.9/34.5
		lab	7.8 ± 6.6 *^,A,B,b^	4.4; 11.2	0.0/8.2/17.3
	HAL-LCU	chair	29.0 ± 9.8 *^,a^	24.0; 33.9	4.6/30.7/40.6
		lab	26.3 ± 9.8 ^B,a^	21.3; 31.3	2.6/28.3/39.7

LED: Light-emitting diode; HAL: halogen; LCU light curing unit; chair: chairside; lab: labside. * No normal distribution. ^A,B^ Significant differences between the PEEK compositions with the same light unit. ^a,b^ Significant differences between the light units with the same PEEK composition.

**Table 3:** Relative frequency of adhesive and cohesive fracture types and 95% CI divided by LCU and PEEK composition.

Fracture Types				
PEEK	Light Curing Unit		Adhesive	Cohesive
PEEK/0%	LED-LCU	chair	100 (80; 100)	0 (0; 19)
		lab	94 (72; 100)	6 (0; 28)
	HAL-LCU	chair	0 (0; 19)	100 (80; 100)
		lab	6 (0; 28)	94 (72; 100)
PEEK/20%	LED-LCU	chair	83 (57; 97)	17 (2; 42)
		lab	100 (80; 100)	0 (0; 19)
	HAL-LCU	chair	0 (0; 19)	100 (80; 100)
		lab	0 (0; 19)	100 (80; 100)
PEEK/>30%	LED-LCU	chair	89 (64; 99)	11 (0; 35)
		lab	89 (64; 99)	11 (0; 35)
	HAL-LCU	chair	11 (0; 35)	89 (64; 99)
		lab	6 (0; 28)	94 (72; 100)

LED: Light-emitting diode; HAL: halogen; LCU light curing unit; chair: chairside; lab: labside.

## Abbreviations

The following abbreviations are used in this manuscript:

PEEK	polyetheretherketone
MMA	methylmethacrylate
DMA	dimethacrlyate
TiO_2_	titanium dioxide
LED	light-emitting diode
HAL	halogen
TBS	tensile bond strength
LCU	light curing unit
FDPs	fixed dental prostheses
VL	visio.link
Al_2_O_3_	alumina oxide
